# The impact of scale and frass recirculation on pathogen inactivation dynamics in black soldier fly larvae bioconversion

**DOI:** 10.3389/fmicb.2025.1539486

**Published:** 2025-03-27

**Authors:** Cecilia Lalander, Ivã Guidini Lopes, Nikos Gyftopoulos, Björn Vinnerås

**Affiliations:** ^1^Department of Energy and Technology, Swedish University of Agricultural Sciences, Uppsala, Sweden; ^2^Department of Biosystems and Technology, Swedish University of Agricultural Sciences, Alnarp, Sweden

**Keywords:** biowaste treatment, eco technology, insect, *Hermetia illucens*, nutrient recycling, waste management

## Abstract

A promising waste management technology that has emerged within the past decade is bioconversion of waste with the larvae of the black soldier fly (BSFL). Regarding waste management purposes, hygiene is central. At present, most studies on BSFL conversion have been performed in small-scale laboratory settings, and the mechanisms behind the documented inactivation of microorganisms remain unclear. In this study, the inactivation dynamics of pathogens and indicator organisms during BSFL bioconversion of food waste was investigated. Two trials were conducted: one mimicking a large-scale industrial setup and another evaluating the impact of frass recirculation on pathogen reduction to better understand the potential role of bioactive compounds in frass. The results indicate that pathogen inactivation observed in small-scale setups is also applicable to large-scale systems, with no significant scale impact on inactivation. The primary difference between scales was increased water evaporation in larger systems, leading to higher electrical conductivity in the frass. Increased solid retention time through frass recirculation did not significantly affect pathogen inactivation but considerably improved the yield of larvae per treated tonne of food waste. The results clearly show that inactivation is linked to larval presence and activity. However, the specific mechanisms driving this effect remain unclear—whether due to bioactive compounds produced by the larvae, physico-chemical changes induced by their activity, or a combination of both. Future research should focus on the microbial risks associated with long-term frass recirculation and further explore the balance between biological and chemical inactivation factors.

## 1 Introduction

Over two billion tonnes of municipal solid waste are generated globally every year, and this is projected to nearly double, reaching almost four billion tonnes annually by 2050 if significant changes are not made regarding how these materials are managed. Although there are large regional differences, the biodegradable fraction of waste (biowaste) constitutes around 50% of the total amount of waste worldwide. In 2020, 38% of the waste generated globally was not handled in any way (uncontrolled handling), whilst 30% of the controlled fraction was landfilled and a mere 19% was recycled (United Nations Environment Programme, 2024). Inadequate management of biowaste can have detrimental impacts on both the environment and public health, leading to greenhouse gas emissions, nutrient leakage into water bodies, and the spread of diseases (Kaza et al., 2018). One reason for the low recycling rate of biowaste is because the cost of transport and treatment generally exceeds the generated revenue (Linden and Reichel, 2020). Biowaste treatment with the larvae of the black soldier fly [BSFL, *Hermetia illucens L.* (Diptera: Stratiomyidae)] has recently emerged as a promising alternative, as two valuable products (larvae and a fertiliser, so-called frass) are generated whilst the treatment complexity can be kept low (Lalander et al., 2018). The produced larval biomass can be used in animal feed (da-Silva et al., 2024), and frass can be used as an organic fertiliser or soil amendment (Lopes et al., 2022). An additional benefit of BSFL biowaste treatment is its ability to improve hygiene by significantly reducing faecal indicators and pathogens such as *Escherichia coli*, *Salmonella* spp., as well as animal viruses. However, it is not a full sanitisation method, as it does not inactivate all disease-causing microorganisms, such as the eggs of the parasitic worm *Ascaris suum* (Lalander et al., 2015).

The natural habitat of BSFL is decomposing organic material (e.g., animal carcasses) containing high quantities of pathogenic microorganisms. A well-developed immune system can thus be considered an advantage for this species, and several bioactive compounds with antimicrobial effects, such as antimicrobial peptides (AMPs), have been extracted from these larvae (Xia et al., 2021). AMPs bind to a pathogen’s cell membrane, disrupting its structure and leading to cell destruction. Gene fragments of AMPs with antimicrobial activity against both gram-negative *E. coli* and gram-positive *Staphylococcus aureus* has been found in in BSFL (Elhag et al., 2017). Both defesin-like and cecropin-like AMPs have been isolated from the hemolymph of immunised BSFL (Park and Yoe, 2017a, 2017b). Moreover, the feed substrate has been shown to impact the inactivation of microorganisms; Vogel et al. (2018) demonstrated a stronger antimicrobial effect against *E. coli* when the larvae where fed a high-protein diet compared to plant oil or lignin-rich diets. This is in accordance with our previous findings, in which reductions in *E. coli* were observed when fish carcasses (containing ~25 mg N-tot g^−1^) were used as a rearing substrate for the larvae (Lopes et al., 2020), but not in a mixture of pig manure, human faeces, and food waste (13 mg N-tot g^−1^) (Lalander et al., 2015). This could be because the BSFL only produce different types of AMPs after an immunisation process, or upon some type of stimuli. Previous findings indicate that some sort of bioactive compounds with antimicrobial effects likely are present in frass, as a reduction in *Salmonella* spp. was observed when it was reinoculated in frass that had previously undergone BSFL composting, whereas no reduction occurred in the same material that had not been processed by BSFL (Lopes et al., 2020). However, the reduction in *Salmonella* spp. in the reinoculated frass was less than that found in the presence of BSFL, which may indicate that these compounds are short-lived and must be continuously generated. Nevertheless, it is possible that the presence of frass as part of the feed substrate provided to the larvae could result in a more pronounced inactivation of pathogens, in case bioactive compounds with antimicrobial effects are present and active in the frass.

Studies investigating pathogen inactivation in BSFL biowaste treatment have only been conducted in laboratory scale conditions that inaccurately reflect the conditions used in the insect industry. Yakti et al. (2022) demonstrated that process efficiency, as well as larval composition, differed when rearing BSFL in small-scale laboratory settings (treatment area of to 200 cm^2^ with 1,200 BSFL) compared to large-scale settings (treatment area close to 2000 cm^2^ with 13,000 BSFL), despite the larval densities and larval feed dose being the same. In certain large-scale insect factories, as many as 60,000 BSFL can be placed in one treatment unit (personal communication). The authors stipulated that the heat formation that occurs when more BSFL aggregate in one part of the treatment unit could play a role, as a temperature difference of more than five degrees was found between the largest and smallest setting. Many parameters are known to impact the process efficiency of waste bioconversion, e.g., substrate composition, larval density, material depth, and larval feeding dose (Lalander and Lopes, 2024). These factors often interrelate, making it difficult to distinguish the impact of one parameter from another (Lopes et al., 2023). Opare et al. (2023) found that the immune response of BSFL (measured by phenoloxidase activity) was impacted by the larval density, with higher larval densities (>5 lv cm^−2^) resulting in a higher immune response. It is unknown whether an immune response equates to production of bioactive compounds with antimicrobial effects.

This study was designed with two main objectives, both with a focus on improving the understanding of pathogen inactivation dynamics in the BSFL conversion process. The first objective was to validate results obtained in a small-scale laboratory setting, for a large-scale setting that mimics common industrial conditions. This step is critical for assessing the scalability and real-world applicability of previous findings. The second objective was to assess the impact of frass recirculation on pathogen inactivation dynamics based on the hypothesis that frass recirculation could change the dynamics of this inactivation, possibly due to the presence of bioactive compounds with antimicrobial effects.

## 2 Materials and methods

### 2.1 Materials

The BSFL used in this study were acquired from a BSF population that has been continuously running since 2015 at the Swedish University of Agricultural Sciences (SLU Uppsala, Sweden). Adult flies are kept in a climate-controlled room (temperature of 30 ± 2°C and humidity set at 50 ± 10%) with egg traps, which are placed on top of a plastic box containing a 1:1 (*w*/*w*) blend of chicken feed (Granngården Hönsfoder Bas with a metabolisable energy content of 10.0 MJ kg^−1^) and water (reaching an approximate moisture level of 70%). Eggs were collected daily, and the newly hatched larvae were reared on this substrate until reaching 1.5–2.0 mg, at which point they were sieved out using a 1–2 mm mesh, and hand counted.

Post-consumer food waste was collected daily for two weeks prior to each trial (described below), at two restaurants located on the SLU campus in Uppsala, and stored in a cold room (4°C). The waste composition was visually different each day, and all inorganic waste materials (plastic, glass, and cutlery) that were identified were manually removed. Subsequently, the waste was milled in a fruit grinder (Voran BG2-5.5 Bio Universal shredder), thoroughly homogenised, and kept at −18°C until use.

The microorganisms used in this study were four serotypes of the Gram-negative bacteria *Salmonella enterica* subspecies Senftenberg, Typhimurium, Typhi, and Dublin, two serotypes of *Escherichia coli*, NCTC 12241 and ATCC 8739, and the bacteriophage φx174, hosted in *E. coli* (ATCC 13706). Bacteria culture and propagation of bacteriophages was carried out in a non-selective bacterial nutrient medium (referred to as *nutrient broth*, Swedish Veterinary Agency, Sweden). Buffered NaCl peptone water with Tween 80 (Swedish Veterinary Agency, Sweden) was used in all dilution series. Xylose lysine desoxycholate agar (XLD) containing 0.15% sodium-novobiocin (Swedish Veterinary Agency, Sweden) was used for *Salmonella* spp. growth, whilst Chromocult®* Coliform Agar (VWR, Sweden) was used for *E. coli* enumeration. For bacteriophage analyses, blood agar base plates (BAB) (Swedish Veterinary Agency, Sweden) were used.

### 2.2 Experimental design

Two trials were conducted in this study. In Trial 1, the dynamics of *Salmonella* spp., *E. coli,* and bacteriophage φx174 were investigated in a system mimicking a large-scale setting, using treatment unit size and process parameters defined by Yakti et al. (2022) as large-scale: treatment units of 60 × 40 × 12 cm (Tofo Trading AB, Sweden); treatment area of 2,400 cm^2^; number of larvae per replicate 12,000; feed provided per replicate 10,5 kg. In Trial 2, the impact of frass recirculation was assessed and only *Salmonella* spp. and *E. coli* were included. For this trial the experimental scale was reduced to smaller treatment units (Smartstore classic with dimension 21 × 11 × 17 cm yielding a treatment area of 231 cm^2^). In both experiments, a larval density of 5 larvae cm^−2^ and a larval feed dose of 0.25 g of total volatile solids (VS) larva^−1^ were adopted. Both trials were conducted in a tent (Secrete Jardin, Hydro Shoot 120; dimensions – 120 × 120 × 200 cm) in which the temperature was regulated to 28.1 ± 2°C using a temperature regulator (Trixie Sverige AB) connected to a heater. The tent was placed inside a microbiology Class 2 laboratory and connected to the building’s ventilation system. The humidity level was monitored throughout the trials and was found to be 28.6 ± 4%. All treatments were performed in triplicates. Further details of each trial are described below.

#### 2.2.1 Trial 1

In Trial 1, the larvae were fed only food waste, which amounted to a total feed load of 10.5 kg per treatment unit and 12,000 BSFL. The food waste was spiked with the four serotypes of *Salmonella* spp., the two *E. coli* serotypes, and the one bacteriophage described in Section 2.1, immediately before being provided to the larvae in a 1% w/w ratio for each microorganism. Different strains of the same microorganism were mixed after selective culture, prior to being inoculated into the food waste. For homogeneous distribution of the inoculant, stepwise mixing was performed in three stages: first with 1% of the material, then with 10%, and lastly with the entire substrate. Three treatments were investigated in this first trial (Table 1): the *control* consisted of food waste without larvae, established for the purpose of observing microbial dynamics in the absence of larvae; *with larvae, two feedings*, in which the total feed load was divided into two feeding events; and *with larvae, three feedings*, in which the total feed load was divided into three feeding events. The treatments with two and three feedings were the same as those explored by Lopes et al. (2020) and were purposefully designed to determine if the distinct dynamics of microbial inactivation observed in the small-scale setup would be similar in a large-scale setup. The treatment units were placed inside the experimental tent in a bakery wagon (ToFo Trading AB, Sweden) with 3 cm spacing between the boxes. The treatment units were relocated within the wagon on every day of sampling to prevent any effects of temperature and ventilation gradients (Johannesdottir, 2017). The trial lasted 11 days, after which the substrate appeared visually processed, and the larvae could be easily and manually sieved from the frass, using 5–10 mm mesh sieves, and each fraction weighed individually. One sample of ten larvae per replicate was weighed to attain a final larval weight. The total mass of larvae was then divided by the final larval weight to estimate the larval survival.

**Table 1:** Components and details of the treatments designed in Trial 1 and Trial 2.

	Food waste	Frass	Contaminated frass	BSFL
Trial 1				
Control	X	-	-	-
With larvae, two feedings	X	-	-	X
With larvae, three feedings	X	-	-	X
Trial 2				
Control	X	-	-	-
Control with 20% frass	X	X	-	-
With larvae, two feedings	X	-	-	X
With larvae, 20% frass	X	X	-	X
With larvae, 20% cont. frass	X	-	X	X

In the treatments containing food waste and frass, the proportion between these two components is 80% food waste and 20% frass, in relation to the feed dose provided to the larvae, on a VS basis.

#### 2.2.2 Trial 2

In Trial 2, larvae were fed either food waste alone or in combination with frass, which was recirculated from previous BSFL bioconversion processes using the same batch of food waste. The frass was used as a dietary component to the BSFL and was produced as described in Lopes et al. (2024). In addition to serving as a dietary component to the BSFL, one hypothesis of the frass recirculation was that anti-microbial peptides or other compounds produced by the BSFL as an immunity response to pathogens, would be present in the frass and thus impact the pathogen inactivation pattern in the BSFL conversion process. Two different types of frasses were used: one originating from a BSFL conversion process conducted with contaminated food waste (*Salmonella* spp. and *E. coli*) and one with non-contaminated food waste. The impact on pathogen dynamics was assessed with both types of frasses to determine if frass that derived from contaminated food waste would lead to an accumulation of either anti-microbial peptides or active pathogenic bacteria. In other words, to determine whether a recirculation of frass would increase or decrease the risk of disease transmission.

##### 2.2.2.1 Production of frass for frass-recirculation

In the first BSFL bioconversion processes in which the frass types were produced, large treatment units (plastic boxes of 60 × 40 × 12 cm, treatment area of 2,400 cm^2^) were used, and placed in bakery wagons (3 cm spacing between boxes) in the experimental tent, as described above. The BSFL conversion procedure was conducted as described by Lopes et al. (2024) and in Section 2.2.1, with a treatment time of 10 d. The larvae were separated from the frass using 5–10 mm mesh sieves.

##### 2.2.2.2 Trial 2 treatments

The second trial was conducted in smaller treatment units, each covered with a lid to prevent the larvae from escaping. A cut-out section (~7 × 4 cm) in the lid was covered with a net to enable air circulation. In the treatments where frass was included in the larvae diet, it constituted 20% of the total larval VS dose, as suggested by Lopes et al. (2024). The total number of larvae added was 1,155 per replicate and the feed dose in all treatments was 0.25 g VS larva^- 1^, similar to Trial 1. Each replicate was placed into a larger treatment box (60 × 40 × 12 cm) and placed in the bakery wagon (3 cm spacing between each larger box) which was placed inside the experimental tent. The food waste was inoculated with *Salmonella* spp. and *E. coli* (bacteriophages were not included in Trial 2), as described in Section 2.2.1. Three treatments and two controls were assessed (Table 1). The control treatments included one control treatment with no frass and no larvae, referred to as *control*, and one *control with frass*, designed to determine whether the potential bioactive compounds with antimicrobial effects in the frass alone could impact the pathogen inactivation dynamics. For all three treatments involving larvae there were two feeding events. In one larval treatment, no frass was recirculated, referred to as *with larvae, two feedings*. In the other two larval treatments, 20% frass was recirculated: one in which 20% non-contaminated frass was recirculated, referred to as *with larvae, 20% frass;* and one in which 20% contaminated frass was recirculated, referred to as *with larvae, 20% cont. frass*. The treatment units were relocated within the wagon on each day of sampling to avoid any effects of temperature and ventilation gradients (Johannesdottir, 2017). The treatment lasted 10 days upon which the larvae were manually sieved from the frass, using 5–10 mm mesh sieves, and each fraction was weighed separately. One sample of ten larvae per replicate were weighed to attain a final larval weight. The total mass of larvae was then divided by the final larval weight to estimate the larval survival.

### 2.3 Microbial inoculum preparation

All bacterial strains used in this study were kept at −80°C. The inoculums (prepared individually for each microorganism and strain) were prepared according to the procedures described in Lopes et al. (2020). Briefly, the bacterial strains and the virus hosted in *E. coli* were cultivated in 5 mL of nutrient broth at 37°C for 2 h at 200 revolutions per minute (rpm), followed by dilution into 45 mL of nutrient broth for 24 h. The concentrated solution was centrifuged (4,500 rpm) and the pellet was dissolved into 50 mL of buffered NaCl peptone water with Tween 80, pH 7.0 (SVA, Sweden). The concentrations of *Salmonella* spp. and *E. coli* achieved in the inoculums were approximately 10^8^ colony forming units (CFU) mL^−1^ and 10^7^ CFU mL^−1^, respectively. For the phage φx174, the inoculum achieved a concentration of 10^8^ plaque forming units (PFU) mL^−1^.

### 2.4 Physico-chemical analyses

Food waste (inflow substrate), frass, and larvae samples were dried in an oven at 65°C for 48 h for determination of total solids (TS). The samples were then combusted in a muffle oven for total volatile solids (VS) determination, following a program in which it took 1 h to reach 250°C which it remained at for 2 h, and then a further hour to reach 550°C, which it remained at for 4 h to ensure proper combustion of all the samples (modified ISO 1822:2015). Food waste and frass samples were analysed for pH and electrical conductivity (EC), by placing 5 g of the materials inside 50-mL Falcon tubes and dissolving it into 20 mL of deionised water, agitating in a vortex mixer for 1 m and after a 1 h resting period, analysing both parameters in a pH meter (InoLab pH meter 913, Metrohm®) and in a conductometer (Conductometer 912, Metrohm®), respectively. Total ammonium nitrogen (TAN) was also analysed from this solution. For that purpose, 1 mL of the solution was collected, diluted in 9 mL of deionised water inside a 15-mL bottle with a small aperture, and had its pH increased to approximately 10 with the addition of 1 mL of a NaOH solution 10 M to convert all ammonium into volatile ammonia (NH_3_). Upon the addition of NaOH, a probe connected to a conductometer was immediately inserted in the aperture of the bottle and TAN was measured in mV.

### 2.5 Microbiological analyses

The concentration of the target microorganisms was assessed on a daily basis (from day 1 to day 11) in Trial 1, whereas in Trial 2 this was accessed on days 1, 2, 3, 5, 8, and 12. The following procedure was adopted: 5 g of the treatment residue were dissolved in 45 mL of Tween buffer and serial dilutions were prepared from this concentrated solution, after a 15-min resting period. Following this, 100 μL of the selected dilution was spread on XLD plates for *Salmonella* spp. enumeration and chromocult plates for *E. coli* enumeration. For sampling of the bacteriophage φx174, the host (*E. coli*) was cultured in a nutrient broth at 37°C for approximately 12 h, at which point 1 mL of the selected dilution was mixed with 2 mL of soft agar and 1 mL of the host solution and then poured onto BAB plates. All microorganisms were incubated at 37°C for approximately 24 h before enumeration.

### 2.6 Calculations

The efficiency of the bioconversion processes was assessed using several commonly applied parameters, including material reduction and bioconversion efficiency (BCE_TS_), on a TS basis and larval survival (%), according to the equations used by Lopes et al. (2024). Additionally, the dynamics of pathogen inactivation were evaluated by means of three parameters: inactivation rate constant (*k*, Equation 1), which gives the log_10_ reduction per time unit; decimal reduction (D_90_, Equation 2), which represents the time (in days) for achieving 1 log_10_ reduction (90%) of the concentration of selected microorganisms; and total logarithmic pathogen reduction (ΔLogRed, Equation 3), which reflects the initial and final concentration of a microorganism throughout the entire experiment. The rate constant *k* was calculated as:


(1) $$k=\frac{\left(\log10\;Nt-\log10\;N0\right)}{\left(Nt-N0\right)}$$

where N_t_ and N_0_ are bacterial concentration at time *t* and at the beginning, respectively.

D_90_ was then calculated as:


(2) $$D90=\left(\frac{-1}{k}\right)$$

ΔLogRed was calculated as:


(3) $$\Delta \mathrm{LogRed}=\log10\left(\frac{{\left[mo\right]}_{t=0}}{{\left[mo\right]}_{t=i}}\right)$$

where [mo]*_t = 0_* is the measured concentration of a selected microorganism in the material at the start (*t* = 0), and [mo]*_t = i_* is the measured concentration of a selected microorganism in the material at time *i*.

### 2.7 Statistical analysis

Analysis of variance (ANOVA) with a 5% significance level (if not otherwise stated) was used to verify statistically significant differences between treatments. In instances where a statistically significant difference was found, Tukey’s honestly significant difference (HSD) *post hoc* multiple comparison test was used to identify which treatments differed. Normality of model residuals was verified using Shapiro-Wilks test with a 5% significance level. In instances where normality was not established (*p* < 0.05), Kruskal-Wallis non-parametric test was used with a 10% significance level, followed by Dunn’s *post hoc* multiple comparison test with *p*-values adjusted with the Benjamini-Hochberg method. A robust linear model (rlm) was applied to evaluate the relationship between the log-transformed concentration, day, and treatment. The robust method was selected because the residuals from the ordinary least squares linear regression model deviated significantly from normality. All statistical analyses and graphical visualisations were performed using R-studio (RStudio Team, 2024). The robust linear model, implemented using the MASS package in R, provided more reliable estimates under these conditions.

## 3 Results

### 3.1 Treatment process

The food waste used in Trial 2 was relatively drier than that used in Trial 1, and it also had a slightly higher VS content (Table 2). The treatment residue after treatment was considerably drier in Trial 1 (large trays) than in Trial 2 (small trays). The larvae in Trial 1 had a higher TS content than those in Trial 2, whilst the VS in the larvae of the different treatments varied with <1%.

**Table 2:** Total solids (TS in %) and total volatile solids (VS, % of TS) of the inflow and outflow materials and the larvae.

	Inflow and outflow materials				Larvae			
	TS (%)		VS (% TS)		TS (%)		VS (% TS)	
	Average^*^	Sd	Average^**^	Sd	Average^*^	Sd	Average	Sd
Inflow materials before treatment								
Trial 1 (*n* = 6)	29.6^a^	1.3	94.6^a^	0.4				
Trial 2 (*n* = 12)	33.7^b,c^	1.1	95.9	0.2				
Outflow materials after treatment								
Control								
Trial 1 (*n* = 3)	41.6^b,c^	1.1	93.9^a^	0.3				
With larvae, two feedings								
Trial 1 (*n* = 3)	64.1^d,e^	4.1	89.0^b^	0.4	39.4^a^	0.2	95.0	0.1
Trial 2 (*n* = 3)	23.6^a^	1.0	91.6	0.2	33.7^b^	2.1	95.4	0.4
With larvae, three feedings (*n* = 3)	74.0^c,e^	1.5	87.2	0.2	41.6^c^	0.3	95.8	0.5
With larvae, 20% frass (*n* = 3)	32.7^a,b,d^	6.8	89.7^b^	0.7	37.9^a,b,c^	0.6	95.6	0.2
With larvae, 20% cont. frass (*n* = 3)	31.1^a,b,d^	1.3	89.7^b^	0.4	38.6^a,b^	0.3	95.8	0.1

Same letters within columns indicates no significant difference (*p* = 0.05 or *p* = 0.1 as specified).

*Kruskal-Wallis non-parametric test, significance level *p* = 0.1.

**ANOVA, significance level *p* = 0.05.

**Figure 1:**
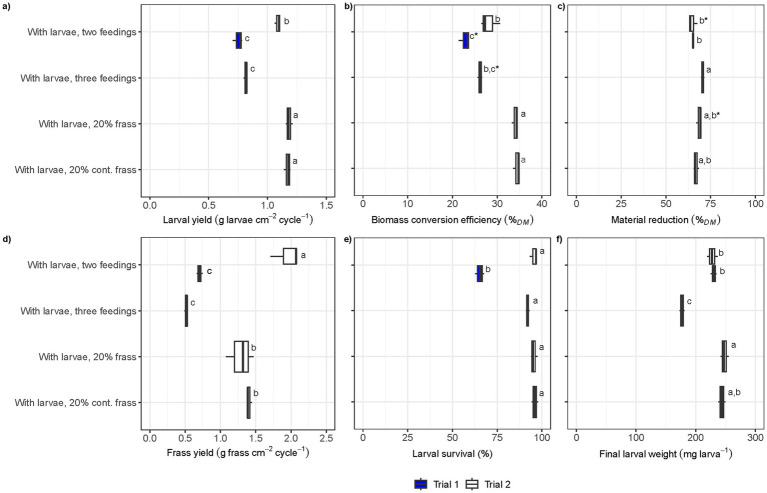
Boxplots of process efficiency parameters in the BSFL conversion process in Trial 1 (blue) and Trial 2 (white): **(a)** larval yield per treatment cycle (g larvae cm^−2^ cycle^−1^), **(b)** biomass conversion efficiency (%*_TS_*), **(c)** material reduction (%*_TS_*), **(d)** frass yield per treatment cycle (g frass cm^−2^ cycle^−1^), **(e)** larval survival (%), and **(f)** final larval weight (mg larva^−1^). Vertical lines in boxplots indicate median, the left edge the 25th percentile and the right edge the 75th percentile. Same letters in the plot indicate no statistical difference (ANOVA, *p* > 0.05). The presence of an asterisk (*) indicates statistical significance at a relaxed threshold (*p* > 0.1).

The treatments with frass inclusion generated the highest larval yield per cm^2^ and cycle (1.1–1.2 g cm^−2^ cycle^−1^), whilst both the treatments with two and three feedings conducted in large trays (Trial 1) had a larval yield <0.85 g cm^−2^ cycle^−1^ (Figure 1a). In fact, the treatment with two feedings conducted in the large trays (Trial 1) resulted in a lower larval yield, biomass conversion efficiency, larval survival, and frass yield compared to the same treatment conducted in small trays with a slightly different food waste (Figures 1a,b,d,e). The treatments involving frass as part of the larvae diets yielded the highest biomass conversion efficiency, reaching 34 ± 1% on a TS basis when including the TS in food waste and the recirculated frass (Figure 1b). This was significantly higher compared to the treatment with two feedings from Trial 2, which had no frass inclusion and achieved 28 ± 2% on a TS basis. The material reduction did not vary greatly between the treatments and trials, yet the frass yield per cm^−2^ and cycle^−1^ was significantly lower in the large tray treatment (<0.75 g frass cm^−2^ cycle^−1^ in Trial 1) (Figure 1c). In the small tray trial (Trial 2), frass yield was significantly smaller in the frass inclusion treatments: 1.3 ± 0.1 g frass cm^−2^ cycle^−1^ in the frass inclusion treatments compared to 2.0 ± 0.2 g frass cm^−2^ cycle^−1^ without frass inclusion (Figure 1d). Larval survival was >90% and did not differ significantly between any of the treatments except the two-feeding treatment in Trial 1, where it was 65 ± 3% (Figure 1e). The final larval weight was <220 mg larva^−1^ in all treatments except the three-feeding treatment in Trial 1 (Figure 1f). The highest larval weight was observed in the frass inclusion treatment (248 ± 7 mg larva^−1^) and the lowest in the three-feeding treatment (177 ± 4 mg larva^−1^).

**Figure 2:**
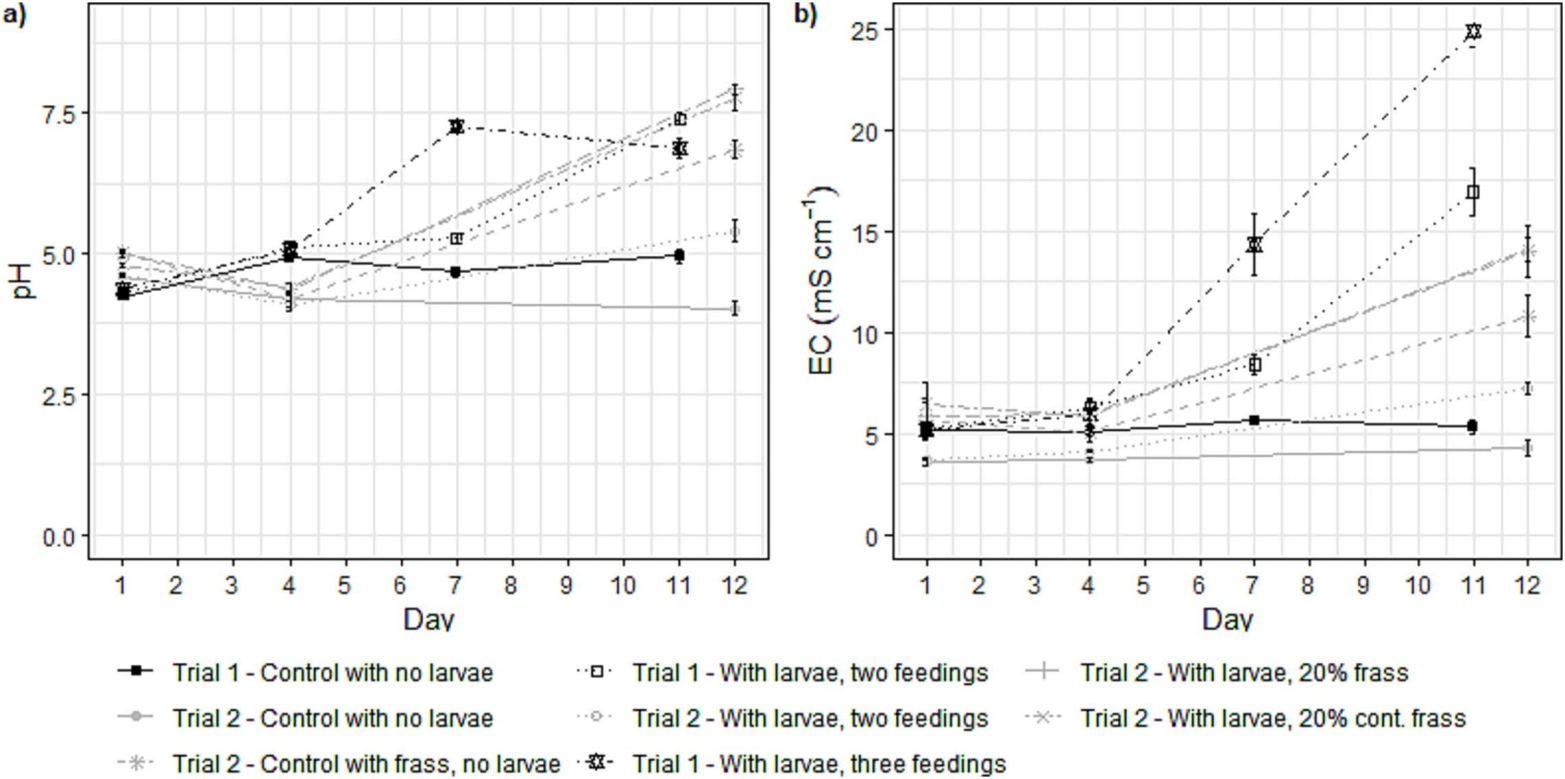
Plots of **(a)** pH and **(b)** electrical conductivity (EC, measured in mS cm^−1^) across different treatments over the duration of the experiment. In Trial 1 (represented in black), measurements were taken on days 1, 4, 7, and 11, and in Trial 2 (represented in grey), measurements occurred on days 1, 4, and 12. Points on the graphs represent mean values (*n* = 3), and the error bars indicate standard deviations.

Although the food waste used in both trials derived from the same two restaurants, the changes observed in pH and EC throughout the bioconversion process differed between the trials. In the first day of both trials, the pH of the food waste was 4.4 ± 0.2 in the absence of frass as part of the substrate and 4.9 ± 0.1 in the presence of frass (Figure 2a). In the absence of larvae, the pH did not vary significantly in both trials, but when larvae were present, the pH increased up to 5.4 ± 0.2 in the absence of frass and up to 7.9 ± 0.1 in the presence of frass. Regarding the EC, larger changes were observed, with the highest EC values being registered at the day of harvest (Figure 2b). In Trial 1, the EC of the digested substrate (frass) reached up to 24.9 ± 0.8 mS cm^−1^, whilst in Trial 2 the highest EC was found in the presence of frass as part of the feed substrate, reaching up to 14.1 ± 0.6 mS cm^−1^.

### 3.2 Inactivation dynamics

**Figure 3:**
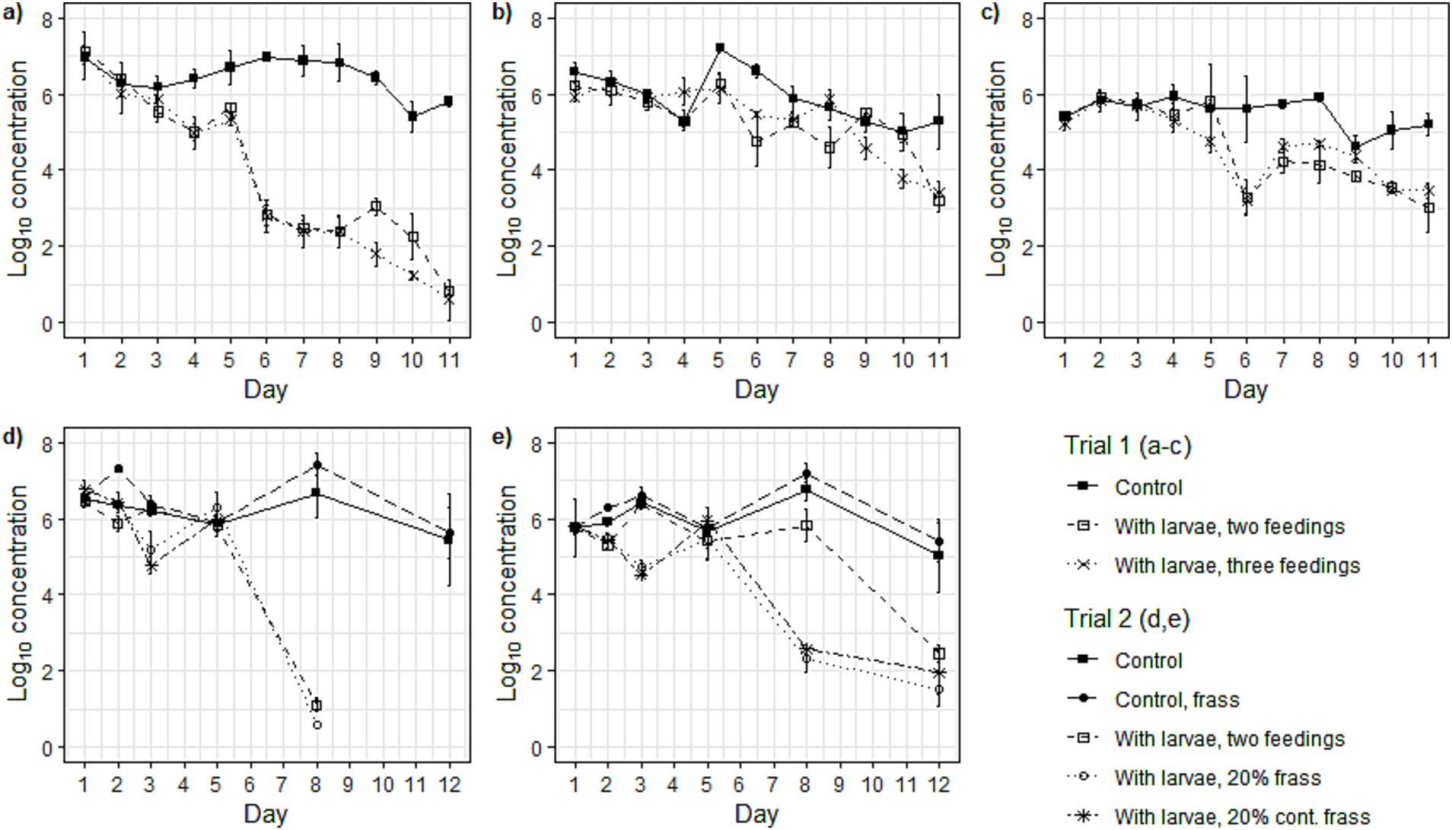
The log_10_ concentration (log_10_ CFU g^−1^) over time in **(a)** *Salmonella* spp., **(b)** *Escherichia coli,* and **(c)** bacteriophage ΦX174 concentrations (log_10_ CFU/PFU g^−1^) in the treatments in Trial 1, and **(d)** *Salmonella* spp. and **(e)** *Escherichia coli* in Trial 2. Points in graphs represents the mean (*n* = 3) and the error bars represent the standard deviation.

Microorganisms were inactivated faster in the treatments than in the controls (without larvae) for all studied microorganisms (Figures 3a–e). The difference compared to the control was greatest in the *Salmonella* spp. concentration over time for the treatments in both Trial 1 (around 2.5 log_10_ difference to the control) and Trial 2 (0.7–1.7 log_10_ difference to control with no larvae) (Table 3). There was no significant difference in the response to the treatments in Trial 1 for any of the studied microorganisms. In Trial 2, there was no significant difference in the treatments’ impact on *Salmonella* spp. concentration over time, however, for *E. coli*, there was a significant difference between the treatment with larvae and two feedings and the treatments with frass inclusion. No differences were found between the two frass treatments for any of the microorganisms. In Trial 2, no significant difference was observed between the control and the control with frass; however, a significant difference was noted between the frass control and all three treatments in the change in concentration over time for the two microorganisms studied (Table 3).

**Table 3:** Summary of the regression models for changes in concentration over time in the treatments [log_10_ (conc) = β0 + β1 x day+β2x treatment] for the studied microorganisms: *Salmonella* spp. and *Escherichia coli* in Trials 1 and 2, as well as the bacteriophage ΦX174 in Trial 1.

	*Salmonella* spp.			*Escherichia coli*			ΦX174		
	Value	*t*-value	*p*-value	Value	*t*-value	*p*-value	Value	*t*-value	*p*-value
Trial 1									
Day	-0.4	−13.1	2 × 10^-16^	−0.2	−10.2	2 × 10^-16^	−0.2	−9.4	3.4 × 10^-15^
Difference to control									
Two feedings	−2.5	−9.4	3 × 10^-15^	−0.6	−4.3	4 × 10^-5^	−0.9	−5.8	8.2 × 10^-8^
Three feedings	−2.8	−10.5	2 × 10^-16^	−0.6	−3.8	2 × 10^-4^	−0.9	−5.6	1.9 × 10^-7^
Difference between treatments									
2 feed: 3 feed			*0.3*			*0.6*			*0.8*
Trial 2									
Day	−0.45	−9.9	10 × 10^-16^	−0.2	−7.3	1 × 10^-10^			
Difference to control									
Control with frass	*0.25*	0.8	*0.4*	*0.24*	0.7	0.5			
Two feedings	*−0.72*	−3.5	8 × 10^-4^	−0.7	−1.9	*0.06*			
Larvae, 20% frass	−1.7	−3.6	6 × 10^-4^	−1.6	−4.5	2 × 10^-5^			
Larvae, 20% cont. frass	−1.5	−3.9	2 × 10^-4^	−1.5	−4.1	8 × 10^-5^			
Difference to control with frass									
Two feedings	−0.42	−4.3	5 × 10^-5^	−0.9	−2.5	0.01			
Larvae, 20% frass	−2.4	−4.4	4 × 10^-5^	−1.9	−5.2	2 × 10^-6^			
Larvae, 20% cont. Frass	−2.5	−4.7	1 × 10^-5^	−1.8	−4.8	7 × 10^-6^			
Difference between treatments									
2 feed: frass			*0.9*			0.01			
2 feed: cont. frass			*0.7*			0.03			
Frass: cont. frass			*0.8*			*0.7*			

The value represents the daily change in log_10_ concentration for the variable “days,” and the total difference in log_10_ concentration between each respective treatment and the control for the variable “treatments.” Non-significant relationships are indicated in italics (*p* > 0.5). *T*-values represent the strength of the observed differences, with larger values indicating stronger evidence against the null hypothesis. The *p*-value gives the probability of obtaining the observed result if the null hypothesis is true.

**Figure 4:**
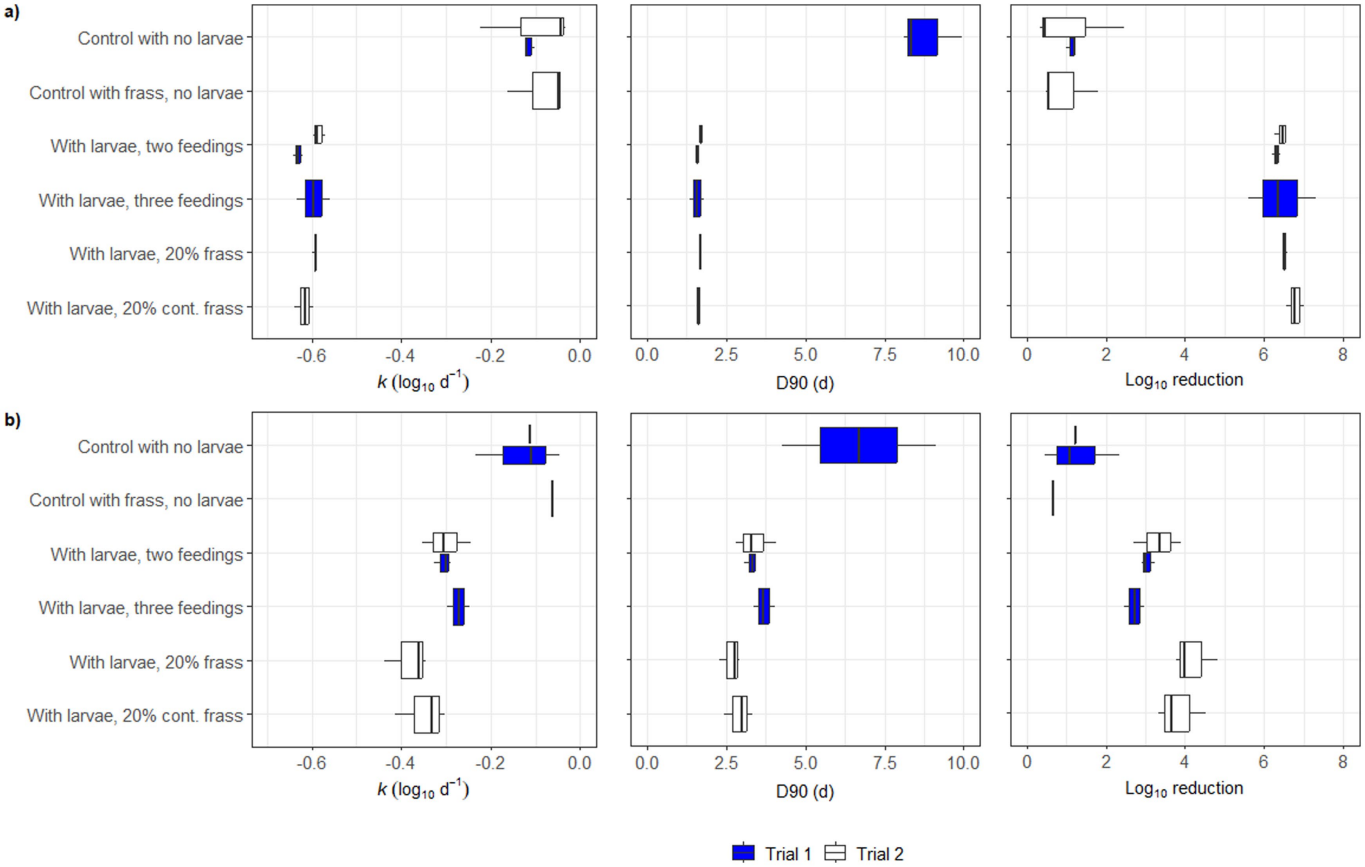
Plots of pathogen reduction parameters k (log_10_ d-1), D90 (d) and total log_10_ reduction in the different treatments in Trial 1 (blue boxes) and Trial 2 (white boxes), for **(a)** Salmonella spp. and **(b)** *Escherichia coli*. Vertical lines in boxplots indicates median, the left edge the 25th percentile and the right edge the 75th percentile. No D90 values were calculated for the controls in Trial 2, as the total reduction over the course of the experiment was less than 90% (1 log_10_).

The inactivation rate (*k*) and log_10_ reductions of both *Salmonella* spp. (Figure 4a) and *E. coli* (Figure 4b) were higher in all treatments than in the controls, whereas there was no difference between the two controls (with and without frass). The *k*-values and log_10_ reductions were higher for *Salmonella* spp. (*k*-values of around −0.6 log_10_ d^−1^ and log_10_ reductions <6 log_10_) in relation to *E. coli* (*k*-values of −0.2 − 0.4 log_10_ d^−1^ and log_10_ reductions 2–4 log_10_). The decimal reduction (D90) was considerably shorter for the treatments compared to the controls (<2.5 d for *Salmonella* spp. and slightly >2.5 d for *E. coli*) and was not calculated for the controls in Trial 2 because the total reduction was not sufficiently large. There was a larger variation in the results of the controls compared to the treatments containing larvae.

Although the total inactivation of *Salmonella* spp. was similar among treatments, with or without the dietary inclusion of frass in Trial 2 (Figures 3, 4), from the first to the eighth day of bioconversion, the concentration of this pathogen differed within treatments. Therefore, the inactivation rate (*k*) was also calculated for other time frames in addition to the total *k* calculated between the first and last day of bioconversion (Supplementary Table S1). Furthermore, between days 1–3 and days 5–8, the rate of inactivation of *Salmonella* spp. was indeed significantly higher in the treatments containing frass (−0.83 ± 0.26 log_10_ d^−1^ and -1.94 ± 0.15 log_10_ d^−1^, respectively) in relation to the control with no larvae (−0.09 ± 0.06 log_10_ d^−1^ and −1.57 ± 0.10 log_10_ d^−1^, respectively).

## 4 Discussion

### 4.1 Process efficiency

There was a significant difference in process efficiencies when comparing the treatment with larvae and two feedings between Trial 1 (large-scale setting) and Trial 2 (laboratory-scale setting) in terms of larval yield, BCE_TS_, frass yield, and larval survival. However, no difference was observed in terms of material reduction and final larval weight (Figure 1). In the large-scale setting, lower larval yield, BCE_TS_, and larval survival were attained. Further, the frass yield was lower, despite the material reduction on a TS basis not being that much higher (Figures 1c,d). This is because more water evaporated in the large-scale setting, indicated by the significantly higher TS content in the outflow material (Table 2). Although all parameters were intended to be kept constant, the food waste used in this experiment came from two separate batches from the same restaurants. The food waste used in Trial 2 had a higher TS and VS than that used in Trial 1 (Table 2). Whilst the composition of the food waste was not analysed (protein, carbohydrates, fat, among other parameters), it could be visually observed that the food waste used in Trial 2 was considerably oilier than that of Trial 1. It has been demonstrated that a varied omnivorous diet, consisting of a balanced mix of proteins, carbohydrates, and fats, shortens larval development times and increases BCE_TS_. In contrast, low-quality substrates with an unbalanced nutrient content lead to longer development times and reduced conversion efficiency (Lalander et al., 2019; Belperio et al., 2024). However, a likely cause of the lower BCE_TS_ and larval yield is the lower larval survival rate that was achieved in Trial 1; around 60% when two feedings were adopted in Trial 1 compared to around 90% in all other treatments. Our results contrast to the findings of Yakti et al. (2022), wherein large-scale settings resulted in a more efficient process in terms of a lower feed conversion ratio (i.e., high substrate-to-biomass conversion efficiency) compared to a small-scale setting. This, the authors stipulated, could be due to the heat, from the aerobic process, produced in the large-scale setting, leading to higher biological activity. It has been previously demonstrated that a feeding scheme involving three-feedings can result in a more efficient process in terms of bioconversion ratio and yield of larval biomass compared to a two-feeding scheme (Lopes et al., 2020). This can be due to the surface of the substrate drying out when feeding twice only, thereby preventing the substrate from being utilised by the BSFL (Lalander et al., 2020). However, a two-feeding scheme may be more economically viable, even though the efficiency is somewhat lower. In Trial 1, the difference in efficiency of three *versus* two feedings was only significant for material reduction, larval survival, and final larval weight. The higher larval survival in the three-feeding scheme seemed to have been counteracted by the smaller final larvae weight, a correlation that has been observed for many substrates (Lopes et al., 2023). The oilier food waste used in Trial 2 could have made the issue of a drying surface less pronounced, even though a two-feeding scheme was used.

Increasing the solids retention time by recycling 20% frass (Haug, 1993) resulted in a small but significant increase in larval yield and a considerable increase BCE_TS_, whilst the frass yield was significantly lower (Figure 1). When Lopes et al. (2024) assessed the impact of incremental increases in frass inclusion as a dietary component on larval yield, BCE_TS_, and material reduction, they found that BCE_TS_ and material reduction decreased with increasing frass inclusion, whereas when analysing the full process the larval yield per kilogram of dry matter food waste (disregarding the frass component of the diet) increased. The pattern observed in this study differed from that reported by Lopes et al. (2024). The BCE_TS_ of the treatment without frass inclusion was higher in this study, at 28 ± 2%, compared to just over 20% in Lopes et al. (2024). Additionally, the treatments with 20% frass inclusion reached a very high BCE_TS_ in this study, 34 ± 1%. When calculating the average larval yield per tonne of food waste on a TS basis for this study, the yield increased from 281 ± 22 kg in the control (no frass recirculation) to 431 ± 8 kg with 20% frass recirculation, with no significant difference observed between uncontaminated and contaminated frass (Supplementary Figure S1). This yield is notably higher than that in Lopes et al. (2024), where it was around 290 kg per tonne at 20% frass inclusion and 400 kg per tonne at 40% inclusion. Although the yield increased with frass inclusion, the rate of increase in this study may be overestimated, as it was based on only two data points. This discrepancy could be attributed to variations in the composition of the food waste, as well as the quality of the recirculated frass. Overall, the process efficiencies achieved in this study indicate that the bioconversion process was highly effective.

The substrate pH of most of the BSFL treatments increased from pH 5 to 6–7.6 (Figure 2a). An increase in pH (up to as high as pH 9) is expected in BSFL bioconversion (Gärttling and Schulz, 2022), as the degradation of macro molecules, such as proteins, leads to the release of ammonia that increases the pH (Parodi et al., 2020). Meneguz et al. (2018) found that the pH reached around 9 in all treatments after around five days of BSFL bioconversion, despite the starting pH being between 4 and 9.5. Lindberg et al. (2022), however, reported that the pH of the frass varied greatly depending on the substrate that the BSFL were reared on, between 3.7 and 10. The reason for the pH increase in Meneguz et al. (2018) could be because it was managed by an addition of NaOH into a Gainesville diet. In Lindberg et al. (2022) the pH was not adjusted and the buffering capacity of the different substrates could thus influence the pH development. The pH of the treatment with larvae and two feedings in Trial 1 increased less than what could be expected, and the frass had a pH just above 5 at the end of the treatment. However, none of the frass attained in Trial 2 were very dry but had a TS content of 34–39%. The pH of the frass was not in the higher range for any of the treatments. Ermolaev et al. (2019) stipulate that a wetter treatment residue will result in a lower pH due to formation of organic acids, something that commonly happen at the start of a composting process (Sundberg, 2005). Additionally, the respiration product CO_2_ can either escape as gas or dissolve into the substrate, forming carbonic acid (H_2_CO_3_), bicarbonate (HCO_3_^−^), and carbonate (CO_3_^2−^), which contributes to pH buffering.

The electrical conductivity (EC) followed a trend similar to that of the pH, with the controls without larvae remaining constant, except for the frass control (Figure 2b). In treatments with BSFL, EC increased, except in the treatment with larvae and two feedings in Trial 2. This increase was expected due to food waste degradation, uptake of ions in the larvae, and loss to the environment through NH_3_, CO_2_ emissions. The incoming food waste had an EC of around 5 mS cm^−1^, and it increased in almost all BSFL treatments and reached 25 mS cm^−1^ in the treatment with larvae and three feedings (Trial 1). Gärttling and Schulz (2022) reported a mean (*n* = 16) EC of frass of 4.03 mS cm^−1^, with a 42% coefficient of variation across multiple frass samples. However, they did not specify which substrates the frass originated from, a factor that likely has a significant impact on the EC. Liu et al. (2018) observed a positive correlation between a reduction in EC and a reduction in extractable Na and extractable K and NH_4_-N when composting poultry litter and related these reductions to a maturation process of the compost. In this case, it could be the opposite, as it has been shown that fresh frass is not mature (Lopes et al., 2024). A clear trend is that the EC in Trial 1 BSFL treatments increased more than those in Trial 2. The considerably lower frass yield observed in Trial 1, due to increased evaporation, likely led to a higher concentration of ions in the frass, thereby explaining the high EC. Generally, a higher EC is not desirable for organic fertilisers that are intended to be applied in the soil, because a higher EC is usually correlated with a higher content of salts which cause phytotoxicity (Hargreaves et al., 2008). In such cases an EC >5 mS cm^−1^ is already considered critical (Vukobratović et al., 2018). Currently, frass is more widely considered as a fertiliser that is added in smaller volumes (Safitri et al., 2024), rather than a growing media such as peat, in which an EC this high would not be acceptable, especially if such a high EC is due to the presence of excessive amounts of salts, which cause significant changes in the soil and inhibit plant growth (Hargreaves et al., 2008).

### 4.2 Pathogen reduction dynamics

In Trial 1, there was no difference in the *Salmonella* spp. inactivation rate when either two or three feeding regimes were adopted (Figures 3a, 4A; Table 3). This contrasts to Lopes et al. (2020), who reported a significantly lower reduction in *Salmonella* spp. when BSFL converted a mixture of fish carcasses and bread using a three-feeding regime compared to a two-feeding regime. In that experiment, the pH was maintained at 7 using a buffer, as pre-trial tests showed that high ammonia concentrations in the substrate inactivated the targeted microorganisms. The frass recirculation did not affect the total salmonella inactivation over the full study time, however, it did appear to increase the rate of inactivation during the first few days of treatment (Figures 3d, 4a; Table 3). The overall reduction in *E. coli* was smaller in Trial 1 than that of Lopes et al. (2020), whilst in Trial 2 it was found to be within the same range (Figures 3b,d, 4B; Table 3). The reduction dynamics of ΦX174 were similar to previous findings in BSFL waste bioconversion (Lalander et al., 2015). The ability of BSFL to inactivate *Salmonella* spp. has been demonstrated in various studies (Erickson et al., 2004; Lalander et al., 2013; Lalander et al., 2015; Lopes et al., 2020). The results on the impact on *E. coli* are less consistent and have been suggested to be dependent on the substrate, as high protein diets can lead to a stronger immune response (Vogel et al., 2018). Nakagawa et al. (2023) demonstrated that the AMP production by the BSFL could be stimulated by either subjecting BSFL to thermal injury or an injection with the gram-positive bacteria *Micrococcus luteus*. Similarly, Opare et al. (2024) demonstrated an increased immune response in BSFL that had been submersed into a solution containing the entomopathogenic fungus *Beauveria bassiana.* Considering that different substrates hosts distinct microbiota (Gold et al., 2020), it could be stipulated that they may stimulate the production of bioactive compounds with antimicrobial effects against specific microorganisms, rather than merely influencing inactivation through differences in nutritional composition.

Another factor that impacts the immune response in BSFL is the larval density as well as the temperature, with higher densities and temperatures resulting in a higher immune response (Opare et al., 2023). This could explain the stronger inactivation that was observed in the large-scale trials for the three-feeding regime (Trial 1) compared to that observed in Lopes et al. (2020), as the density was higher in the present study (5 lv cm^−2^ compared to 4 lv cm^−2^) and the temperature in the larger boxes can be expected to be higher than that of the smaller boxes (Yakti et al., 2022). The faster inactivation observed in the treatments with frass as part of the larvae diet (Supplementary Table S1) could be due to bioactive compounds already being present in the substrate when the experiment began. Still, the presence of these compounds alone could not inactivate the investigated microorganisms, as there was no significant difference in inactivation dynamics between the control and the control with frass (Table 3). Lopes et al. (2020) demonstrated that inactivation could occur in the frass without the presence of BSFL when re-inoculating the frass with *Salmonella* spp. and *E. coli.* Conversely, in this experiment, no inactivation was observed in the frass control with no larvae, suggesting that the concentration of bioactive compounds may have been too low for inactivation when only 20% of the substrate was frass. This suggests that the presence and activity of the BSFL is necessary for the effect of these compounds to have an impact when the concentration is lower. Thus, recirculating the frass seems to improve or at least maintain the overall hygiene of the process rather than worsen it. If a more persistent microbial agent that is not inactivated or destroyed in the BSFL bioconversion is present (such as the eggs of parasitic worms), there is a risk that these would be accumulated in the process with frass recirculation. Future studies should investigate the effect of higher inclusions of frass in the larvae diet, in relation to the inactivation of pathogens, to verify if this possible inactivation is dose dependent. Moreover, the impact of age (or time of storage) of frass on the inactivation dynamics should be studied.

The third factor that could influence the inactivation of intestinal bacteria and animal viruses is the production of bicarbonate (HCO_3_^−^) (Sundberg, 2005) and ammonia (NH_3_) (Beck-Friis et al., 2003) during the degradation of food waste, both of which significantly impact microorganism survival (Vinnerås et al., 2008; Dobay et al., 2018). The effect of ammonia is closely linked to pH, as only certain forms exhibit antimicrobial properties, as the uncharged form of ammonia becomes more prevalent at a high pH and this form is more effective at inactivating bacteria by disrupting intracellular pH (Kohn et al., 2017). Additionally, ammonia can inactivate bacteriophages by damaging their genetic material, further contributing to its antimicrobial effect (Oishi et al., 2016). Unlike ammonia that is highly pH-dependent, bicarbonate concentrations directly influence cellular activity by regulating cAMP levels, which in turn suppresses protein synthesis (Xie et al., 2010). Variations in treatment efficiency can be attributed to fluctuations in parameters such as temperature and pH. Higher process temperatures increase cell permeability and chemical reactivity, amplifying the antimicrobial effects (Cruz-Loya et al., 2021).

A variety of factors influence the survival of contaminating microorganisms, with the substrate playing the most significant role as it influences which metabolites that are produced during the process, including both simple molecules such as ammonia and more complex bioactive compounds. The addition of frass initially impacted the *k*-value, indicating some reduction in microbial load. However, no significant difference in the survival of *Salmonella* spp. or *E. coli* could be observed throughout the study in correlation with the circulation of frass. No significant inactivation in the studied microorganisms occurred in the frass control without larvae (Figure 3), demonstrating that the frass inclusion alone did not have any impact on the inactivation dynamics. Had the effect been purely chemical, on the other hand, a broader range of microorganisms would likely be impacted than what was observed for the BSFL treatment. For example, the eggs of *Ascaris suum* have been shown to be sensitive to ammonia treatment (Nordin et al., 2009), yet helminth eggs are unaffected by the BSFL bioconversion process (Lalander et al., 2013). Likewise, the inactivation of *E. coli* would likely have been greater than what was observed in this study (Nordin, 2010). Bioactive compounds, such as antimicrobial peptides, are generally species-specific, aligning well with the pathogen dynamics observed in this study. Larval activity was clearly vital for pathogen inactivation; however, the exact mechanism behind this process remains unclear. Although our data suggest that bioactive compounds may play a key role, we did not verify their presence. Additionally, we cannot rule out that physico-chemical changes induced by larval activity also contribute to the observed inactivation. Further studies are needed to better understand the mechanisms involved, as well as to identify the specific compounds responsible.

## 5 Conclusion

In this study, we investigated the inactivation dynamics of indicator organisms and pathogens during the BSFL conversion process. Our findings confirmed that the pathogen inactivation observed in small-scale settings is also applicable to larger-scale systems. No significant scale effect was observed on inactivation, apart from greater water evaporation in the large-scale setting, which led to higher electrical conductivity (EC) in the frass. Increasing the solids retention time through internal recirculation of frass did not significantly impact pathogen inactivation but greatly improved the yield of larvae per tonne of food waste. Our findings indicate that larval activity is crucial for pathogen inactivation, though the exact mechanisms remain unclear. While bioactive compounds appear to play a key role, their presence was not verified, and physico-chemical changes caused by larval activity cannot be ruled out. Further research is needed to clarify the underlying processes and identify the specific compounds, alongside assessing microbial risks associated with long-term frass recirculation, including risks from both human and insect pathogens.

## Data Availability

The raw data supporting the conclusions of this article will be made available by the authors, without undue reservation.
