# Supplementary material for: The impact of scale and frass recirculation on pathogen inactivation dynamics in black soldier fly larvae bioconversion
Source: Front Microbiol. 2025 Mar 27;16:1539486. doi: 10.3389/fmicb.2025.1539486 (PMC11984946; doi:10.3389/fmicb.2025.1539486)
Supplement: Supplementary file 1 [file Data_Sheet_1.DOCX]

**Supplementary information**

# The impact of scale and frass recirculation on pathogen inactivation dynamics in black soldier fly larvae bioconversion

Cecilia Lalander^a,^*, Ivã Guidini Lopes^b^, Nikos Gyftopoulos^a^ and Björn Vinnerås^a^

^a^ Department of Energy and Technology, Swedish University of Agricultural Sciences, Uppsala, Sweden

^b^ Department of Biosystems and Technology, Swedish University of Agricultural Sciences, Alnarp, Sweden

**Corresponding author*

E-mail address: [cecilia.lalander@slu.se](mailto:cecilia.lalander@slu.se)

Inactivation rates were calculated for different time spans: days 1–3, 5–8, 5–12, and 1–2 (Table S1).

Table S1. Inactivation rate constant (k, calculated according to Eq. 1, given in log_10_ d^-1^) calculated for distinct time frames (from first to third day; fifth to eighth day; fifth to twelfth day; and from the first to the twelfth day) throughout Trial 2.

|  | *k*_days 1-3_ | *k*_days 5-8_ | *k*_days 5-12_ | *k*_days 1-12_ |
| --- | --- | --- | --- | --- |
|  | *Salmonella* spp. | | | |
| Control | -0.160 ± 0.046 | 0.263 ± 0.184 | -0.062 ± 0.162 | -0.099 ± 0.088 |
| Control, frass | -0.090 ± 0.122 | -1.179 ± 1.123 | -0.038 ± 0.099 | -0.085 ± 0.055 |
| With larvae, two feedings | -0.094 ±0.045 | -1.574 ± 0.079 | -0.832 ± 0.034 | -0.585 ± 0.011 |
| With larvae, 20% frass | -0.675 ± 0.225 | -1.881 ± 0.174 | -0.901 ± 0.043 | -0.593 ± 0.003 |
| With larvae, 20% cont. frass | -1.003 ± 0.078 | -1.991 ± 0.031 | -0.853 ± 0.013 | -0.616 ± 0.016 |
|  | *Escherichia coli* | | | |
| Control | 0.328 ± 0.049 | 0.390 ±0.073 | -0.089 ± 0.113 | -0.066 ± 0.064 |
| Control, frass | 0.414 ± 0.110 | 0.482 ± 0.139 | -0.053 ± 0.007 | -0.035 ± 0.036 |
| With larvae, two feedings | 0.315 ± 0.361 | 0.136 ± 0.071 | -0.423 ± 0.069 | -0.301 ± 0.044 |
| With larvae, 20% frass | -0.499 ± 0.105 | -1.046 ± 0.205 | -0.560 ± 0.115 | -0.381 ± 0.040 |
| With larvae, 20% cont. frass | -0.627 ± 0.031 | -1.120 ± 0.059 | -0.570 ± 0.063 | -0.349 ± 0.046 |

Significant higher larval yield per tonne of food waste was achieved when 20% frass was recirculated as feed component in the larval diet (Figure S1).


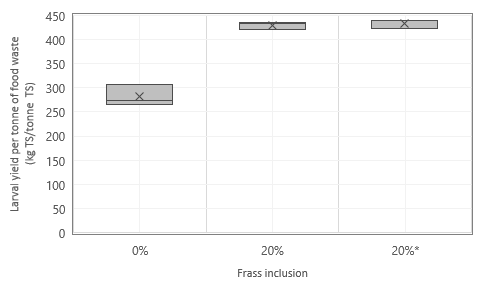


Figure S1. Box plot showing the larval yield per tonne of food waste on a TS basis (kg TS/tonne TS) for three treatments with varying frass inclusion levels: 0%, 20%, and 20%* (contaminated frass). The boxes represent the interquartile range (IQR), with the lower and upper boundaries indicating the 25th and 75th percentiles, respectively.
